# Coping methods of college students with different personality traits when facing COVID-19 from the anxiety psychology perspective

**DOI:** 10.3389/fpsyg.2024.1357225

**Published:** 2024-05-30

**Authors:** Hai Fu, Yuyao Mao, Li Tian

**Affiliations:** ^1^School of Liberal Arts, Nantong University, Nantong, Jiangsu, China; ^2^School of Humanities and Communication, Ningbo University, Ningbo, Zhejiang, China

**Keywords:** personality traits, coping styles, college students, anxiety, COVID-19

## Abstract

**Objective:**

Based on a survey of anxiety among college students during the epidemic, this study takes anxiety as an example to study the coping methods of college students with different personality traits. Thus predicting the behavioral tendencies of college students and proposing some appropriate suggestions for the current psychological education work of college students. Method: The study was carried out during the large-scale outbreak of the COVID-19 epidemic, and the investigation lasted one month. Using the Self Rating Anxiety Scale (SAS), Eysenck Personality Questionnaire Simplified Chinese Version (EPQ-RSC), and Trait Coping Style Scale (TCSQ), an online questionnaire survey was conducted on 932 college students to analyze the mutual effects of different grades, genders, personality traits, coping methods, and other factors.

**Results:**

The research found that there was a significant gender difference in negative coping methods and anxiety among college students. Grade differences: In the comparative study of personality traits, there are gender differences in introversion and concealment dimensions and grade differences in neuroticism and concealment dimensions. There is a pairwise correlation between personality traits, coping methods, and anxiety. There is a significant positive correlation between the dimensions of psychoticism, neuroticism and anxiety; There is a significant negative correlation between introversion, concealment, and anxiety. Positive coping methods are significantly negatively correlated with anxiety, while negative coping methods are significantly positively correlated with anxiety. The positive coping style is significantly negatively correlated with the dimensions of psychoticism and neuroticism and positively correlated with the dimensions of introversion, introversion, and concealment; Negative coping methods are significantly positively correlated with the dimensions of psychoticism and neuroticism and negatively correlated with the dimensions of introversion, introversion, and concealment.

**Conclusion:**

The research results indicate that the mental health issues of college students need to be taken seriously to prevent the spread of anxiety.

## 1 Introduction

Coronavirus Disease 2019 (COVID-19) has gradually broken out around the world since the end of 2019, undoubtedly causing a considerable impact and seriously endangering people’s physical and mental health. According to the data released by the World Health Organization on May 4, as of April 2023, the cumulative number of COVID-19 infections worldwide has exceeded 765 million, with more than 6.9 million deaths. Many studies have attempted to investigate how COVID-19 affects people’s mental health in the short and long term. Some studies suggest that outbreaks of COVID-19 can have serious mental health effects, with increased symptoms of depression, anxiety, insomnia, and acute stress (Dragioti et al., 2022). At the same time, in addition to the troubles caused by the epidemic, relevant prevention and control measures have also directly or indirectly affected people’s lives. The isolation measures taken in response to the epidemic have had a significant impact on the mental health of the population (Di Giuseppe et al., 2020). The research found that after one month of the blockade, people’s psychological stress will increase, especially among women and young people (Niedzwiedz et al., 2020). Affected by COVID-19, college students will be more vulnerable in terms of mental health. A study compared the clinical symptoms of two groups of college students seeking psychological intervention in the university psychological counseling center before and after the COVID-19 pandemic, and found that COVID-19 had a significant negative impact on them (Cerutti et al., 2022). During the most severe period of the COVID-19 epidemic, prevention and control measures mainly focused on home isolation and wearing masks when traveling. While blocking the spread of the virus, it also had a significant impact on the learning and life of college students. In the pre-containment period, the prevalence and incidence rate of common mental disorders (CMD) among British adults has increased (Chandola et al., 2022). For many people, the level of loneliness during the containment period is very high (Bu et al., 2020). In the Netherlands, especially during the winter of 2021, the most stringent blockade period, depression and anxiety symptoms will increase significantly; Blockades have increased symptoms of depression and anxiety among people (Mangot-Sala et al., 2023).

During the COVID-19 pandemic, the public is highly susceptible to a series of adverse psychological stress reactions such as anxiety, worry, and pessimism, which vary from physical and mental health to work and life. This is especially true for students, as the impact of the epidemic and prevention and control measures on their learning and daily lives is unavoidable. Surveys have shown that over 90% of students worldwide are affected by the epidemic in their everyday learning. Moreover, to some extent, their mental health will also be affected, and there may even be hidden dangers left behind. Research shows that depression and anxiety symptoms of adolescents and young people increase during COVID-19 (Hawes et al., 2022). Depression and anxiety symptoms of adolescents also continue to increase (Cochran et al., 2023). A cross-sectional experimental study found that during isolation, the most common symptoms in primary and secondary schools were anxiety (24.9%), depression (19.7%), and stress (15.2%) (Tang et al., 2021). Lifestyle changes, such as school closures, isolation, and the risk of infection at any time, are significantly associated with depression and anxiety in children and adolescents (Meherali et al., 2021). Due to the recurrence and uncertainty of the epidemic, colleges and universities have taken rigorous isolation and protection measures to ensure the safety of college students. In the face of the problematic epidemic situation and strict sealing and control management, college students are prone to stress and psychological contradictions in all aspects of life. Their mental health is generally on the decline (Chao and Fan, 2021), and which is prone to anxiety, fear, worry, and other negative emotions. A study on Bangladeshi school adolescents found that the prevalence rates of moderate to severe depression and anxiety were 37.3 and 21.7%, respectively (Koly et al., 2023). The other study also suggested that it was important to view college students as a psychologically disadvantaged group and provide them with specific assistance (Cerutti et al., 2023). Thus, the psychological status of students deserves attention. In addition to facing the uncertainty of exams and enrollment arrangements and the shift in learning styles, college students face various maladaptations brought about by lockdown management due to the long duration of the epidemic prevention and control front line. This situation is almost unprecedented. In the context of rapid online information dissemination, college students quickly receive information about the epidemic, making them highly susceptible to various negative information influences. To some extent, they may develop some negative emotions and even have psychological health problems as a result (Liu, 2020). Secondly, while worrying about the epidemic, college students must focus on their studies or consider the future. For those who are about to graduate and face employment pressure in their senior year, research has shown that after the outbreak of the epidemic, nearly half of college students have increased their employment anxiety due to the epidemic. College students will feel pressure to participate in activities such as communication, learning, and graduation ceremonies while experiencing difficulties and anxiety about employment issues.

Therefore, it is crucial to pay timely attention to students’ mental and psychological health and help them cope with the problematic environment healthily and appropriately. Coping “refers to the constantly changing cognitive and behavioral efforts individuals make to address specific internal and external environmental requirements that they perceive as beyond their ability and resources (Lazarus and Folkman, 1984). Coping style, also known as coping style, refers to a series of cognitive evaluation and action strategies (Labrague et al., 2018) adopted by individuals to alleviate internal tension and balance their psychological state when they are in a specific external or internal stress state, playing a significant regulatory role in psychological stress. Different coping methods can lead to different psychological stress reactions. Positive coping methods tend to adopt positive actions and strategies to change and alleviate stress (Ding et al., 2015), while negative coping methods are coping methods centered around negative evaluation and emotional expression, avoidance of stress, and social isolation (Chen, 2016). The psychological hazards caused by the same difficulties or encounters often vary from person to person. Some studies believe that this may be related to coping methods. The survey found that during COVID-19, there were differences in coping methods among college students of different sexes and grades, and some coping methods were related to anxiety (Zhao, 2021). Some researchers also believe that personality traits have a significant impact, and some studies have found that adult Iranian populations have lower scores of extroversion, responsibility, and agreeableness, which are risk factors for anxiety and depression (Nouri et al., 2019). Personality traits have predictive value for psychological problems (stress, anxiety, and depression) (Alizadeh et al., 2018). A study targeting lung cancer patients found that anxiety is closely related to personality traits and coping methods (Shimizu et al., 2015). The same holds in the context of the epidemic. Research suggests that anxiety, depression, and personality traits are closely related (Ren et al., 2005). Depression and stress among home quarantine personnel are negatively correlated with the E and L dimensions of the Eysenck Personality Questionnaire and positively correlated with the P and N dimensions (Zhang et al., 2020).

To sum up, in the face of various problems in life caused by various learning and lifestyle changes under the epidemic situation, college students will inevitably have psychological stress and psychological problems. If not effectively alleviated, it will affect the mental health of college students. Therefore, we need to pay attention to this situation and conduct in-depth investigation and research to help everyone alleviate psychological problems and restore a healthy mental state in time. The reduction in the scope and living space of interpersonal communication among college students, the conflicts and contradictions that arise when spending more time with roommates, and the inconvenience caused by long-term dormitory classes are all more likely to trigger anxiety. However, due to the persistent and widespread behavioral tendencies exhibited by personality traits, different individuals possess different traits. However, individuals with the same trait may exhibit relatively consistent responses and behaviors. There are also differences in the coping methods of different individuals. Therefore, the purpose of this study is to investigate the anxiety of college students and study the coping style of students with different personality traits in the case of the COVID-19 outbreak so as to predict the behavior tendencies of college students and put forward some appropriate suggestions for the current psychological education of college students.

This study proposes the following assumptions:

*Hypothesis 1*: College students’ personality traits, coping methods, and anxiety are different in demographic variables.

*Hypothesis 2*: There is a correlation between personality traits and anxiety among college students.

*Hypothesis 3*: There is a correlation between coping methods and anxiety among college students.

*Hypothesis 4*: There is a correlation between personality traits and coping methods among college students.

## 2 Research methods

### 2.1 Subjects and procedures

Randomly select students from a full-time university in China as participants, including grades one to four, and conduct a questionnaire survey. In China’s education system, grade usually refers to the learning stage of students in school. Universities usually have a four-year program. As the sampling time is in the period of strict prevention and control of COVID-19, students do not leave their dormitories unnecessarily. The questionnaire is compiled and collected through a survey platform called Question Star and distributed online to the respondents through WeChat, QQ, and other social software. An IP address can only answer the questionnaire once, and the questionnaire can be submitted after all items are filled in.

According to the usage standards of each scale, invalid questionnaires were excluded, and 932 valid questionnaires were ultimately collected, including 312 female students and 620 male students. There were 195 freshmen, 643 sophomores, 43 juniors, and 51 seniors, among which mean self-evaluation anxiety score under each variable showed a significant increase compared to the domestic norm (29.78 ± 0.46) (see Table 1).

**Table 1:** Distribution of participants (*n* = 932).

Name	Options	Frequency	Percentage (%)
Sex (*n* = 932)	Female	312	33.48
	Male	620	66.52
Grade (*n* = 932)	Freshman	195	20.92
	Sophomore	643	68.99
	Junior	43	4.61
	Senior	51	5.47
Total		932	100

### 2.2 Survey tools

#### 2.2.1 Self-rating anxiety scale

The Self-Rating Anxiety Scale was initially developed by Zung of Duke University School of Medicine, which can intuitively reflect people’s subjective feelings of anxiety (Zung, 1971). This scale consists of 20 survey items used to reflect the subjective feelings of anxiety patients. It includes 5 reverse scoring items and 15 positive scoring items, each using a factor score of 1–4 points. Add the scores of 20 items together to obtain the total SAS rough score (*X*). Multiply the rough score by 1.25 and take the integer part to obtain the SAS standard score (*Y*). According to the results of the Chinese norm, a total score of 50 points or less indicates no anxiety symptoms, a score of 50–59 indicates mild anxiety, a score of 60–69 indicates moderate anxiety and a score of over 69 indicates severe anxiety. Cronbach’s of this scale α the coefficient is 0.920.

#### 2.2.2 Eysenck personality questionnaire simplified Chinese version

Eysenck personality questionnaire (EPQ) is a questionnaire to measure personality dimensions, which was prepared by the famous personality psychologist and clinical psychologist Professor Eysenck, H. J. & Eysenck, S.B.G. It is one of the most commonly used personality questionnaires in China, widely used in healthcare, education, scientific research, and personnel, and has broad application value and prospects. The Chinese version of the Eysenck Personality Questionnaire (EPQ) was revised based on Eysenck’s personality (Qian et al., 2000). It consists of four subscales: Introversion and Extroversion Scale E, Nerve Quality Scale N, Mental Quality Scale P, and Concealment Scale L, including 48 items. Its reliability and validity are reliable.

#### 2.2.3 Trait coping style questionnaire

The Trait Coping Style Questionnaire is used to evaluate individuals’ relatively stable coping strategies toward various events in life and has a certain degree of reliability and validity (Jiang and Zhu, 1999). This questionnaire consists of two dimensions, negative coping and positive coping, each containing 10 items. It is evaluated on a 5-level scale from “definitely yes” to “definitely not.” The higher the scores of PC and NC, the higher the level of coping. This scale actively responds to Cronbach’s α. The coefficient is 0.89, indicating a negative response to Cronbach’s α. The coefficient is 0.78, which has good reliability.

### 2.3 Statistical methods

We described our sampling plan, all data exclusions (if any), all procedures, and all measures taken in the study, and followed the method checklist in the Journal of Applied Psychology. All data, analysis code, and research materials can be obtained in the stable blocking link to the repository. This study used Excel to desensitize, clean, and organize the extracted data, resulting in descriptive statistics, multivariate analysis of variance, correlation analysis, regression analysis, and mixed path analysis using the SPSS 22.0 statistical software AMOS24.0 (Analyze of Moment Structures), to explain the relationship between anxiety levels, personality traits, and coping methods based on the observed results. All data has been publicly available in the APA repository on the Open Science Framework (OSF) And can be accessed at [OSF | Coping methods of College Students with Different Personality Traits, Registrations].

## 3 Results

The overall situation of coping methods and anxiety among college students during the lockdown period of the epidemic is shown in Table 2.

**Table 2:** Coping style and anxiety status of college students (M ± SD).

Project	M ± SD
Actively respond	35.338 ± 6.706
Negative coping	25.754 ± 7.770
Anxiety self-assessment	35.929 ± 9.813

The detection rate of anxiety symptoms among college students is 9.33%; The rates for grades 1 to 4 are 1.07, 33.3, 9.3, and 15.6%, respectively. The results of Table 2 indicate that positive coping *M* = 35.338 is between 30.22 ± 8.72 in the average score of the general population, negative coping *M* = 25.754 is between 23.58 ± 8.41 in the average score of the general population and anxiety score *M* = 35.929. The overall anxiety status is normal.

The results of the independent sample *T*-test (as shown in Table 3) show that anxiety scores of college students of different genders show significant differences (t = 2.367, *p* = 0.018 < 0.05), and the specific comparative differences indicate that the female average (37.00) will be significantly higher than the male average (35.39).

**Table 3:** Differences in anxiety levels between different genders.

		*n*	M ± SD	*t*	*df*	*p*
Anxiety standard score	Female	312	37.00 ± 9.40	2.367	930	0.018*
	Male	620	35.39 ± 9.98			

*p < 0.05, **p < 0.01. The following are all the same.

The results of one-way ANOVA (as shown in Table 4) showed significant differences in anxiety scores among college students of different grades (*F* = 8.244, *p* = 0.000 < 0.05), indicating differences.

**Table 4:** Differences in anxiety levels among different grades.

		*n*	M ± SD	*f*	*df*	*p*
Anxiety	Freshman	195	33.94 ± 8.12	8.244	930	0.018*
	Sophomore	643	35.97 ± 9.91			
	Junior	43	38.40 ± 10.61			
	Senior	51	40.96 ± 11.67			

The results of the post-test in Table 5 show that there were significant differences in anxiety scores among college students of different grades, with the lowest level of anxiety in the first year and the highest level of anxiety in the fourth year.

**Table 5:** Post-test of anxiety scores among college students of different grades (mean difference).

	Freshman and sophomore	Freshman and junior	Freshman and senior	Sophomore and junior	Sophomore and senior	Junior and senior
Anxiety	−2.034	−4.465*	−7.02**	2.431	−4.986**	−2.555

The results of the independent sample *T*-test (as shown in Table 6) indicate that college students of different genders do not show significant differences in positive coping methods (*p* > 0.05), but show significant differences in negative coping methods (*t* = 5.473, *p* = 0.000 < 0.05), and the average value of women (27.60) is significantly higher than that of men (24.82).

**Table 6:** Differences in coping methods between different genders.

	Female (*n* = 312)	Male (*n* = 620)	*t*	*p*
	(M ± SD)	(M ± SD)		
Positive coping score	35.71 ± 5.78	35.15 ± 7.12	1.281	0.201
Negative coping score	27.60 ± 6.94	24.82 ± 8.00	5.473	0.000**

The results of one-way ANOVA (as shown in Table 7) indicate that different grades of college students do not show significant differences in positive coping methods (*p* > 0.05). However, there is a significant difference in negative coping methods (*F* = 6.299, *p* = 0.000 < 0.05), which requires *post hoc* analysis.

**Table 7:** Differences in coping methods among different grades.

	Freshman (*n* = 195)	Sophomore (*n* = 643)	Junior (*n* = 43)	Senior (*n* = 51)	*F*	*p*
	(M ± SD)	(M ± SD)	(M ± SD)	(M ± SD)		
Positive coping score	36.35 ± 6.07	35.14 ± 6.86	34.49 ± 7.78	34.69 ± 5.90	2.061	0.104
Negative coping score	25.31 ± 7.66	25.42 ± 7.75	28.26 ± 7.10	29.57 ± 7.73	6.299	0.000**

The post-test results in Table 8 indicate significant differences in negative coping methods among college students of different grades, with the most significant differences being between seniors and freshmen, and seniors and sophomores.

**Table 8:** Post-test of negative coping methods among college students of different grades (mean difference).

	Freshman and sophomore	Freshman and junior	Freshman and senior	Sophomore and junior	Sophomore and senior	Junior and senior
Negative coping methods	−0.112	−2.948	−4.261**	2.836	−4.149**	−1.313

From Table 9, it can be seen that there is a significant negative correlation between positive coping and anxiety (*r* = −0.446, *p* < 0.01). There is a significant positive correlation between negative coping and anxiety (*r* = 0.465, *p* < 0.01).

**Table 9:** Correlation study between coping methods and anxiety levels of college students.

	Positive coping score	Negative coping score
Anxiety	−0.446**	0.465**

The results of the independent sample T-test (as shown in Table 10) indicate that in terms of personality differences among college students of different genders, the average score of male college students is higher than that of female college students in the dimension of spirituality; In the dimension of Neuroticism, the average score of female college students was higher than that of male college students, but there was no significant difference (*p* > 0.05). There were significant differences in introversion and concealment dimensions (*t* = 2.958, *p* = 0.003 < 0.05; *t* = −2.010, *p* = 0.045 < 0.05). In this introversion dimension, the average value for women (51.79) was significantly higher than that for men (49.53). Regarding concealing this dimension, the female average (52.76) is significantly lower than the male average (54.24).

**Table 10:** Differences in personality traits of different genders in various dimensions.

	Female (*n* = 312)	Male (*n* = 620)	*t*	*p*
	(M ± SD)	(M ± SD)		
Psychosis P	47.02 ± 8.91	47.98 ± 9.26	−1.505	0.133
Inclination E	51.79 ± 10.29	49.53 ± 11.31	2.958	0.003**
Neuroticism N	47.62 ± 11.64	47.28 ± 10.94	0.432	0.666
Cover up score L	52.76 ± 9.96	54.24 ± 10.95	−2.01	0.045*

The results of one-way ANOVA (as shown in Table 11) showed that different grades of college students did not show significant differences in terms of psychoticism and introversion (*p* > 0.05). However, there are significant differences in Neuroticism and concealment dimensions (*F* = 4.33, *p* = 0.005 < 0.05; *F* = 6.25, *p* = 0.000 < 0.05), which requires post-test analysis.

**Table 11:** Differences in various dimensions of personality traits among different grades.

	Freshman (*n* = 195)	Sophomore (*n* = 643)	Junior (*n* = 43)	Senior (*n* = 51)	*F*	*p*
	(M ± SD)	(M ± SD)	(M ± SD)	(M ± SD)		
Psychoticism	47.12 ± 8.48	47.50 ± 9.29	49.94 ± 8.31	49.69 ± 10.21	2.02	0.11
Inward and outward inclination	51.99 ± 10.84	49.86 ± 11.22	49.85 ± 9.04	49.62 ± 10.39	1.96	0.118
Neuroticism	46.43 ± 10.58	47.17 ± 11.07	49.37 ± 12.15	52.29 ± 12.70	4.33	0.005**
Conceal score	53.85 ± 10.90	54.35 ± 10.53	50.16 ± 9.23	48.69 ± 10.62	6.25	0.000**

The post-test results in Table 12 show significant differences in Neuroticism and concealment dimensions among college students of different grades. Among them, senior students scored the highest in the Neuroticism dimension and the lowest in the concealment dimension, with the most significant differences between freshmen and sophomores.

**Table 12:** Post-test of neuroticism and concealment of college students of different grades (mean difference).

	Freshman and sophomore	Freshman and junior	Freshman and senior	Sophomore and junior	Sophomore and senior	Junior and senior
Neuroticism	−0.734	−2.935	−5.861**	2.201	−5.127**	−2.925
Conceal	−0.497	3.694	5.159*	−4.19	5.655**	1.465

### 3.1 A study on the relationship between personality traits and anxiety

Table 13 shows that there is a significant positive correlation between the two dimensions of psychoticism and Neuroticism in personality traits and anxiety (*r* = 0.251, *p* < 0.01; *r* = 0.524, *p* < 0.01); There was a significant negative correlation between introversion, concealment, and anxiety (*r* = −0.258, *p* < 0.01; *r* = −0.253, *p* < 0.01).

**Table 13:** Correlation study between personality traits and anxiety levels of college students.

	Psychotic	Introversion and extroversion	Neuroticism	Conceal
Anxiety standard score	0.251**	−0.258**	0.524**	−0.253**

It can be seen from Table 14 that positive coping has a significant negative correlation with the two dimensions of personality traits, psychoticism and Neuroticism (*r* = −0.270, *p* < 0.01; *r* = −0.383, *p* < 0.01), and a significant positive correlation with introversion and introversion and concealment (*r* = 0.385, *p* < 0.01; *r* = 0.199, *p* < 0.01). There was a significant positive correlation between negative coping and the two dimensions of personality traits: psychoticism and Neuroticism (*r* = 0.101, *p* < 0.01; *r* = 0.537, *p* < 0.01), and a significant negative correlation between negative coping and introversion and concealment (*r* = −0.322, *p* < 0.01; *r* = −0.388, *p* < 0.01).

**Table 14:** Research on the relationship between personality traits and coping methods of college students.

	Positive coping score	Negative coping score
Psychoticism	−0.270**	0.101**
Inward and outward inclination	0.385**	−0.322**
Neuroticism	−0.383**	0.537**
Conceal	0.199**	−0.388**

Perform linear regression analysis using anxiety as the dependent variable and various factors of personality traits as independent variables.

It can be seen from Table 15 that the model formula is: anxiety score = 14.153 + 0.158 psychoticism P-0.111 introversion and extroversion+0.418 Neuroticism, *R*^2^ = 0.319, personality traits can explain 31.9% variation of anxiety score. Psychoticism and Neuroticism can have a significant positive impact on anxiety; The introverted and introverted dimensions have a significant negative impact on anxiety.

**Table 15:** Regression analysis of personality traits and anxiety among college students.

Predicted variable	Predicting variable	β	*t*	*R* ^2^	*R* ^2^_adj_	*F*
Anxiety	Neuroticism	0.158	5.265**	0.319	0.317	144.899
	Inward and outward inclination	−0.111	−4.413**			
	Psychoticism	0.418	17.111**			

Linear regression analysis used positive and negative coping scores as independent variables and anxiety as dependent variables.

According to Table 16, the model formula is anxiety score = 42.021–0.524 positive coping score+0.483 negative coping score, *R*^2^ = 0.338. Negative coping has a significant positive impact on anxiety, while positive coping has a significant negative impact on anxiety. Both can explain the 33.8% variance in anxiety scores.

**Table 16:** Regression analysis of coping methods and anxiety among college students.

Predicted variable	Predicting variable	β	*t*	*R* ^2^	*R* ^2^_adj_	*F*
Anxiety standard score	Positive coping score	−0.524	−13.055**	0.338	0.336	236.821
	Negative coping score	0.483	13.93**			

Linear regression analysis was conducted using various factors of personality traits as independent variables and coping methods as dependent variables.

According to Table 17, the regression equation is:

**Table 17:** Regression analysis of personality traits on coping methods.

Predicted variable	Predicting variable	β	*t*	*R* ^2^	*R* ^2^_adj_	*F*
Positive coping score	Psychoticism	−0.114	−5.328**	0.267	0.265	112.92
	Inward and outward inclination	0.174	9.737**			
	Neuroticism	−0.181	−10.43**			
Negative coping score	Psychoticism	−0.025	−1.082**	0.336	0.334	156.695
	Inward and outward inclination	−0.161	−8.187**			
	Neuroticism	0.344	17.997**			

Positive coping score = 40.565–0.114 psychotic+0.174 introversion and extroversion −0.181 Neuroticism, *R*^2^ = 0.267;

Negative coping score = 18.788–0.025 psychoticism −0.161 introversion and extroversion+0.344 Neuroticism, *R*^2^ = 0.336;

Personality traits can explain 26.7% of the variance in positive coping and 33.6% in negative coping.

Introversion and introversion have a significant positive impact on positive coping and a significant negative impact on negative coping; Neuroticism will have a significant negative impact on positive coping and a significant positive impact on negative coping. Psychoticism will only have a significant negative impact on positive coping.

### 3.2 A path model of personality traits, coping methods, and anxiety levels in college students

Through the above discussion of regression analysis, it can be seen that there is a pairwise correlation between anxiety, personality traits, and coping methods in specific dimensions, indicating that there may be a mediating effect between the three. The following is a mixed path analysis of AMOS to test the variables further under the premise of pairwise correlation among the three.

**Figure 1:**
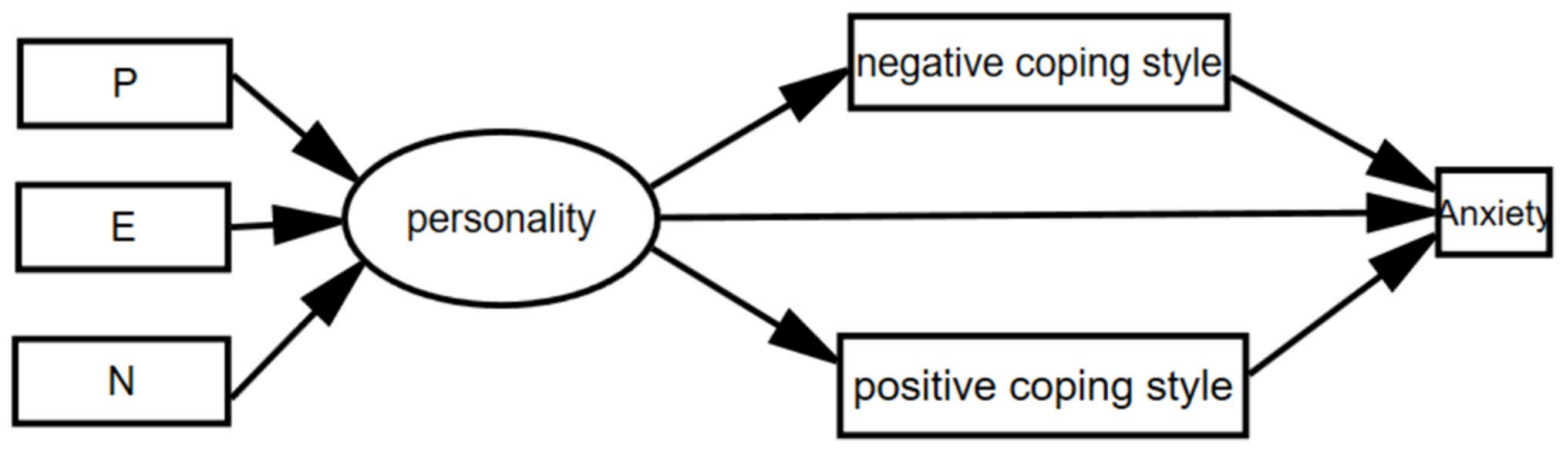
The constructed structural equation model.

Propose the following hypothesis: (1) College students’ coping methods (including positive and negative) directly affect the level of anxiety. (2) College students’ coping methods (including positive and negative) play a mediating role between their personality traits (including neuroticism, spirituality, introversion and extroversion) and anxiety levels. (3) Personality traits of college students (including neuroticism, spirituality, and introversion) indirectly affect the level of anxiety. Based on the above assumptions, the constructed structural equation model is shown in Figure 1.

The model was estimated using the maximum likelihood method, but the path coefficients were insignificant and removed. Reconstruct the model and ultimately conclude that positive coping methods among college students play a partial mediating role between the introverted and introverted dimensions of personality traits and anxiety levels. Using Model 4 of the PROCESS macro program in SPSS, conduct 5,000 self-sampling tests, namely the mediation effect test.

As shown in Table 18, the Bootstrap 95% CI of the mediating effect does not include 0 ([−0.18, −0.11]), indicating a significant mediating effect.

**Table 18:** Analysis of the mediating effects of positive coping methods.

	Effect value	Standard error	Bootstrap 95%CI		Ratio to the total effect
			Lower limit	Upper limit	
Total effect	−0.23	0.03	−0.28	−0.17	60.80%
Direct effect	−0.09	0.03	−0.14	−0.03	
Indirect effect	−0.14	0.02	−0.18	−0.11	

*p < 0.05, **p < 0.01.

**Figure 2:**
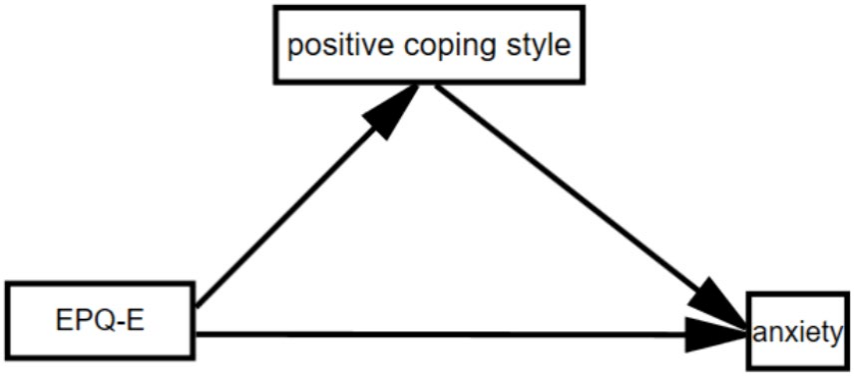
The mediating pathway diagram.

The mediating pathway diagram is shown in Figure 2. Therefore, the upbeat coping style of college students plays a partial mediating role between the introversion dimension of personality traits and the level of anxiety, with the mediating effect accounting for 60.8% of the total effect.

## 4 Discussion

### 4.1 Analysis of personality traits and coping style characteristics of college students

Through comparison, it was found that there are specific gender differences in the personality traits of college students. The female students are higher than the male students in introversion and extroversion and lower than the male students in the dimension of concealment, and the difference is statistically significant. There is a difference, but not significant, in the dimensions of psychoticism and Neuroticism. Male students are extroverted, introverted, and polarized, leaning toward maturity and sophistication, while female students have characteristics such as being outgoing, sociable, emotionally exposed, and lively. The results may vary from previous studies due to different geographical locations, questionnaire choices, and target populations. In the dimension of Neuroticism, there are also significant differences among college students of different grades. Senior students have higher scores in the dimension of Neuroticism. Some senior students are close to employment, and their future is uncertain, especially during the epidemic; they will encounter more significant pressure. Freshmen face more problems of adaptability. Compared with senior students, they face less pressure and confusion. This may be the reason why senior students are more volatile.

Research has shown that there are significant differences in negative coping methods among college students in terms of gender and grade. Compared to boys, girls may have more problems using coping mechanisms. Among them are physiological reasons and social environment factors, such as the subtle influence of traditional social concepts, which may also be related to the parents’ different family Parenting styles for children from childhood to adulthood and society’s different requirements for different gender roles. Suppose parents adopt obedient and indulgent Parenting styles for children from childhood. In that case, children are more likely to use immature coping methods to deal with various problems in life. Male college students often receive more attention, and parents and their surroundings often place more expectations and favors on them. Moreover, society has higher demands on men, and the gender stereotypes conveyed by various aspects of society can have varying degrees of impact. Fourth-grade students also showed significantly higher levels of negative coping methods than freshmen and sophomores. During the pandemic, college students have been under lockdown and management, making it almost impossible to leave school. However, first-year students are curious about the campus and have a fresh sense of life. Second-year students have already adapted to life under the epidemic for a year and may not feel too anxious or bored about not being able to leave school. For senior students, on the one hand, many exams are being postponed, and on the other hand, it is challenging to intern or find a job outside the school. In addition, the epidemic has also increased the difficulty of finding a job. For example, job fairs can only be held online, and various exams are postponed or held online, exacerbating the difficulty of finding a job. They are facing employment pressure and are more likely to experience situations such as insomnia and panic and suddenly face difficult situations. Lack of coping experience, they are unable to adjust themselves promptly and stabilize their emotions, so they tend to adopt more damaging ways to cope. However, the scores of senior students in the Neuroticism dimension are on the high side, which may also be affected by personality traits.

Therefore, it is necessary to guide college students to handle problems more actively and adjust their mindset based on gender characteristics, different grades, and different personality traits.

### 4.2 Analysis of anxiety characteristics of college students

The data of this study shows that there are significant differences in anxiety between genders and grades, with females significantly higher than males. Female students’ anxiety emotions are more affected during the epidemic, which is consistent with other research findings (Niedzwiedz et al., 2020). At the same time, freshmen have the lowest level of anxiety, while seniors have the highest level of anxiety, possibly influenced by coping methods. Female college students have significantly higher levels of negative coping methods than male college students, and seniors have significantly higher levels of negative coping methods than freshmen and sophomores. This may, to some extent, affect their handling of psychological problems and troubles when facing the epidemic and lockdown; negative coping is more likely to make it difficult for them to resolve their accumulated negative emotions, leading to the possibility of psychological symptoms.

### 4.3 A study on the relationship between personality traits and coping methods

There are varying degrees of correlation between the scores of college students’ coping methods and various factors of personality, personality traits and anxiety, and coping methods and anxiety. A cross-sectional study showed that during the pandemic when college students were studying online at home, their negative emotions were influenced by Optimal personality tension (Wei et al., 2023). Another study found that lifestyle, healthy behavior, and appropriate coping strategies can all alleviate mental health symptoms (Zhang et al., 2023), which is consistent with the results of this study. In this study, there is a significant correlation between positive and negative coping methods and the P, E, N, and L dimensions of personality traits. This indicates that college students with different personality traits tend to choose different coping methods to face unexpected events and close troubles during the epidemic. The analysis of mediating effects shows that positive coping methods play a partial mediating role between personality traits’ introversion and extraversion dimensions and anxiety levels, indicating the importance of positive coping methods.

Introversion and extroversion are the differences that individuals exhibit in introversion and extraversion, which may lead to irritability, impulsiveness, and a tendency to take risks and socialize well. Introverted individuals are easily influenced by their surroundings and form conditioned reflexes. The research results indicate that the introverted and introverted dimensions have a significant negative impact on anxiety. Extroverted individuals are less susceptible to environmental influences and are more independent and outgoing. When facing problems, they are also more inclined to choose positive coping methods to take positive actions, such as being willing to seek help, communicating with others to resolve problems, or alleviating stress, resulting in lower anxiety levels. However, students with higher levels of spirituality tend to be single, find it challenging to adapt to the environment, use less favorable coping methods, and have difficulty dealing with anxiety issues. Students with a high Neuroticism dimension are likelier to worry or have strong emotional reactions. Their coping style is affected by Neuroticism, which will make them tend to use negative coping methods. Research shows that there is a significant positive correlation between negative coping and anxiety. In the face of emergencies during the epidemic, they are more likely to worry or conflict with others but find it difficult to cope during isolation, so much so that contradictions accumulate in the heart and cannot be adjusted, and psychological problems become increasingly severe. On the contrary, when college students with low scores in the Neuroticism dimension encounter stimuli, their emotional response is slow, and it is easier to recover to a stable emotional state quickly. In this case, individuals are more likely to make objective and positive judgments in the face of pressure, which makes them more inclined to adopt positive coping methods, and are relatively less affected.

By studying the coping strategies of college students with different personality traits, a new perspective has been provided to promote their active response to large-scale public health emergencies. However, the research is limited by geographical and human resources, and the sampling is limited to one university. Based solely on anxiety surveys, the understanding of negative psychological emotions among college students is not comprehensive enough. In the future, multiple methods such as random sampling, expanding the sample size, including multiple scales, and behavioral observation can be used for further research.

### 4.4 Recommendations

The epidemic of novel coronavirus pneumonia has been effectively controlled through the joint efforts of increasingly improved medical mechanisms and the masses. However, a few cases will still appear in some regions as time passes, and universities must be alert at any time. For college students who are in an essential stage of physical and mental development, sudden public health events can easily affect them, especially psychological distress such as irritability and anxiety, and generate certain negative emotions. Therefore, universities must arrange targeted mental health protection work for college students. Therefore, based on the conclusions of this study, the following recommendations are proposed:

1. The anxiety of female college students and fourth-grade students will be more affected during the pandemic, and they should be given more attention, guidance, and intervention promptly. It is recommended to develop relevant psychological counseling based on gender characteristics and different grades of students and provide college students with more help in adaptability. Firstly, schools can improve the level of scientific epidemic prevention awareness among college students by conducting scientific popularization lectures on epidemic prevention knowledge. Secondly, during the employment stage, efforts should be made to pay attention to the current situation of employment anxiety among college students, especially those who exhibit severe anxiety. Timely psychological counseling should be provided to help them overcome employment anxiety and reduce physical symptoms caused by employment anxiety through professional employment psychological counseling, dynamically grasp the employment anxiety status of college students, and try to alleviate the employment anxiety psychology of senior students as much as possible.
2. By conducting mental health courses such as emotional management and distributing psychological assessments, it is possible to pay more attention to students with excessively introverted personalities and poor emotional stability when carrying out mental health education for college students. They should pay attention to their psychological status and receive psychological intervention as soon as problems are discovered while preventing the occurrence of psychological problems. Through specific guidance and training, we aim to help them improve some negative traits in their personalities as much as possible, care about their inner troubles and psychological problems, timely help them overcome negative emotions, face difficulties with a positive and upward attitude, or use methods such as psychological counseling hotlines or remote psychological service platforms to help college students in need relieve their emotions. However, personality traits are gradually formed through the interaction between biological inheritance and parental rearing, and compared to other qualities, they will be more stable and less prone to change. Therefore, coping strategies may be a better entry point.
3. Actively use artificial intelligence to detect and promptly alleviate anxiety or depression among college students. For example, they are using artificial intelligence technologies such as natural language processing (NLP) and machine learning (ML) to conduct emotional analysis on the language of college students, such as social media posts and school assignments, to identify signs and severity of negative emotions such as anxiety and depression. By utilizing the generative dialog function of large language models, a positive and supportive dialog environment can be created; please Encourage students to express their feelings and seek help actively. For example, a chat robot can be developed as a mental health supporter for students, providing listening and guidance anytime. Through dialog with students, artificial intelligence can monitor emotional changes in real time and remind students or professionals to take intervention measures when abnormal situations are discovered. However, it should be noted that although artificial intelligence has great potential for application, it cannot replace the role of professional psychological counselors. Professional psychological counseling or treatment should be provided for students who require in-depth support and intervention.
4. Develop mature coping strategies tailored to the characteristics of different populations. Coping strategies can help regulate people’s anxiety and depression symptoms during the second wave of the COVID-19 pandemic, and interventions that promote positive coping styles can help reduce the negative psychological emotions caused by the pandemic (Miola et al., 2023). College students with different personality traits and genders exhibit different coping methods, which may be related to their families, growth experiences, and social environment. It is necessary to pay more attention and analyze and understand the actual background and specific situation to help them recognize psychological problems in a targeted manner. For example, for college students with high scores in the Neuroticism dimension, their emotions tend to be volatile, which affects their coping style to a certain extent. Through online psychological counseling or timely contact with class psychological committee members, psychological counselors, dormitory leaders, and other classmates, we can maintain communication with them. Students can help each other and help them promptly choose appropriate ways of venting, adjusting, and balancing their emotions, thereby reducing the frequency of using harmful coping methods. We can promote epidemic prevention knowledge to college students based on their characteristics in a timely manner, guide them to maintain a good mindset and rational thinking, teach them to seek help appropriately, alleviate emotional pressure, etc. In that case, it can help them adopt correct and positive methods to alleviate negative emotions. School mental health education courses can also give students more attention and support based on specific situations, such as helping them develop positive coping methods through group counseling. Cultivating the use of appropriate positive coping methods among college students can help improve their ability to face stress, even if they encounter similar or even different situations after entering society. College students who have learned positive coping methods can have the ability and mentality to solve conflicts and stress.
5. Under the current situation of normalization of COVID-19, the school should start from many aspects and do a good job of psychological health protection for college students. For example, conducting regular evaluations of the mental health status of students in order to identify and solve their psychological problems promptly; Regularly organizing mental health-themed class meetings, psychological drama performances, mental health knowledge competitions, and other activities to enhance students’ interest and participation in mental health.

## 5 Conclusion

This study is based on a survey of anxiety among college students during the epidemic and investigates the coping methods of college students with different personality traits. Analyze and study the mutual influence of factors such as different grades, gender, personality traits, and coping methods among 932 college students. Conclusion: Personality traits and coping strategies have a certain predictive effect on the overall anxiety of college students. The mental health issues of college students need to be given more attention and attention by schools and mature coping strategies should be developed according to the characteristics of different groups of college students.

## Data availability statement

The raw data supporting the conclusions of this article will be made available by the authors, without undue reservation.

## Ethics statement

The studies involving humans were approved by Nantong University Ethics Committee. The studies were conducted in accordance with the local legislation and institutional requirements. The participants provided their written informed consent to participate in this study.

## Author contributions

HF: Conceptualization, Data curation, Formal analysis, Funding acquisition, Investigation, Methodology, Project administration, Resources, Software, Supervision, Validation, Visualization, Writing – original draft, Writing – review & editing. YM: Data curation, Formal analysis, Investigation, Methodology, Resources, Software, Validation, Visualization, Writing – original draft. LT: Conceptualization, Data curation, Writing – review & editing, Project administration, Validation, Resources.

## References

[ref1] Alizadeh Z. Feizi A. Rejali M. Afshar H. Keshteli A. H. Adibi P. (2018). The predictive value of personality traits for psychological problems (stress, anxiety and depression): results from a large population-based study. J. Epidemiol. Glob. Health 8, 124–133. doi: 10.1016/j.jegh.2017.11.003, PMID: 30864753 PMC7377556

[ref2] Bu F. Steptoe A. Fancourt D. (2020). Loneliness during a strict lockdown: trajectories and predictors during the COVID-19 pandemic in 38, 217 United Kingdom adults. Soc. Sci. Med. 265:265, 113521. doi: 10.1016/j.socscimed.2020.113521, PMID: 33257177 PMC7768183

[ref3] Cerutti R. Spensieri V. Amendola S. Biuso G. S. Renzi A. Tambelli R. (2023). Responding to the COVID-19 public health emergency: the usefulness of an online brief psychological intervention with Italian university students[J]. Psychol. Sch. 60, 1499–1513. doi: 10.1002/pits.22785, PMID: 36246432 PMC9538663

[ref4] Cerutti R Spensieri V Amendola S. Clinical symptoms in pre-COVID 19 pandemic versus COVID 19 pandemic samples of Italian university students. (2022).

[ref5] Chandola T. Kumari M. Booker C. L. Benzeval M. (2022). The mental health impact of COVID-19 and lockdown-related stressors among adults in the UK. Psychol. Med. 52:2997. doi: 10.1017/S0033291720005048, PMID: 33280639 PMC7783135

[ref6] Chao B. Fan J. (2021). Study on mental health problems and countermeasures of college students under the background of epidemic prevention and control. Psychiatr. Danub. 33, 646–650. doi: 10.24869/psyd.2021.646 34928924

[ref7] Chen C. (2016). The role of resilience and coping methods in subjective well-being among Chinese university students. Asia Pac. Educ. Res. 25, 377–387. doi: 10.1007/s40299-016-0274-5

[ref8] Cochran G. Cohen Z. P. Paulus M. P. Tsuchiyagaito A. Kirlic N. (2023). Sustained increase in depression and anxiety among psychiatrically healthy adolescents during late stage COVID-19 pandemic. Front. Psych. 14:1137842. doi: 10.3389/fpsyt.2023.1137842, PMID: 37009105 PMC10063786

[ref9] Di Giuseppe M. Zilcha-Mano S. Prout T. A. Perry J. C. Orrù G. Conversano C. (2020). Psychological impact of coronavirus disease 2019 among Italians during the first week of lockdown. Front. Psych. 11:576597. doi: 10.3389/fpsyt.2020.576597, PMID: 33192713 PMC7554332

[ref10] Ding Y. Yang Y. Yang X. Zhang T. Qiu X. He X.. (2015). The mediating role of coping style in the relationship between psychological capital and burnout among Chinese nurses. PLoS One 10:e0122128. doi: 10.1371/journal.pone.0122128, PMID: 25898257 PMC4405204

[ref11] Dragioti E. Li H. Tsitsas G. Lee K. H. Choi J. Kim J.. (2022). A large-scale meta-analytic atlas of mental health problems prevalence during the COVID-19 early pandemic. J. Med. Virol. 94, 1935–1949. doi: 10.1002/jmv.27549, PMID: 34958144 PMC9015528

[ref13] Hawes M. T. Szenczy A. K. Klein D. N. Hajcak G. Nelson B. D. (2022). Increases in depression and anxiety symptoms in adolescents and young adults during the COVID-19 pandemic. Psychol. Med. 52, 3222–3230. doi: 10.1017/S0033291720005358, PMID: 33436120 PMC7844180

[ref14] Jiang Q. Zhu Y. (1999). Further exploration of trait response questionnaire. Chin. Behav. Med. Sci. 8, 167–169,

[ref15] Koly K. N. Islam M. S. Potenza M. N. Mahumud R. A. Islam M. S. Uddin M. S.. (2023). Psychosocial health of school-going adolescents during the COVID-19 pandemic: findings from a nationwide survey in Bangladesh. PLoS One 18:e0283374. doi: 10.1371/journal.pone.0283374, PMID: 36972260 PMC10042372

[ref16] Labrague L. J. McEnroe-Petitte D. M. Leocadio M. C. Van Bogaert P. Cummings G. G. (2018). Stress and ways of coping among nurse managers: an integrative review. J. Clin. Nurs. 27, 1346–1359. doi: 10.1111/jocn.14165, PMID: 29148110

[ref17] Lazarus R. S. Folkman S. (1984). Stress, appraisal, and coping. Berlin: Springer Publishing Company.

[ref19] Liu X. (2020). Psychological problems of college students during the isolation period of COVID-19 epidemic and the intervention countermeasures: a case study of Hubei second Normal university. J. Hubei Univ. Econ. 17, 131–134,

[ref20] Mangot-Sala L. Smidt N. Liefbroer A. C. (2023). Changes in anxiety and depression symptoms during the Covid-19 lockdown in the Netherlands. The moderating role of pre-existing mental health, employment situation and alcohol consumption. Soc. Psychiatry Psychiatr. Epidemiol. 58, 1561–1571. Advanced online publication. doi: 10.1007/s00127-023-02480-6, PMID: 37024616 PMC10079151

[ref21] Meherali S. Punjani N. Louie-Poon S. Abdul Rahim K. Das J. K. Salam R. A.. (2021). Mental health of children and adolescents amidst COVID-19 and past pandemics: a rapid systematic review. Int. J. Environ. Res. Public Health 18:3432. doi: 10.3390/ijerph18073432, PMID: 33810225 PMC8038056

[ref22] Miola A. Caiolo S. Pontoni G. Pozzan E. Moriglia C. Simionato F.. (2023). Anxiety and depression during the second wave of the COVID-19 pandemic: the role of coping strategies. Int. J. Environ. Res. Public Health 20:2974. doi: 10.3390/ijerph20042974, PMID: 36833670 PMC9957361

[ref23] Niedzwiedz C. L. Green M. J. Benzeval M. Campbell D. Craig P. Demou E.. (2020). Mental health and health behaviours before and during the initial phase of the COVID-19 lockdown: longitudinal analyses of the UK household longitudinal study. J. Epidemiol. Community Health 75:231:jech-2020-215060. doi: 10.1136/jech-2020-215060, PMID: 32978210 PMC7892383

[ref24] Nouri F. Feizi A. Keshteli A. H. Roohafza H. Afshar H. Adibi P. (2019). Personality traits are differently associated with depression and anxiety: evidence from applying bivariate multiple binary logistic regression on a large sample of general adults. Psychiatr. Danub. 31, 448–456. doi: 10.24869/psyd.2019.448, PMID: 31698401

[ref25] Qian M. Wu G. Zhu R. Zhang S. (2000). Revision of the Eysenck personality questionnaire simplified Chinese version (EPQ-RSC). J. Psychol. 3, 317–323,

[ref26] Ren H. Yang X. Zhang J. Liu M. Gong H. Liu S. (2005). The correlation between anxiety and depression among medical students and their personality traits. Chin. School Health 26:953,

[ref28] Shimizu K. Nakaya N. Saito-Nakaya K. Akechi T. Ogawa A. Fujisawa D.. (2015). Personality traits and coping methods explain anxiety in lung cancer patients to a greater extent than other factors. Jpn. J. Clin. Oncol. 45, 456–463. doi: 10.1093/jjco/hyv024, PMID: 25762799

[ref29] Tang S. Xiang M. Cheung T. Xiang Y. T. (2021). Mental health and its correlates among children and adolescents during COVID-19 school closure: the importance of parent-child discussion. J. Affect. Disord. 279, 353–360. doi: 10.1016/j.jad.2020.10.016, PMID: 33099049 PMC7550131

[ref30] Wei J. Yun Z. Zhang Y. Mei X. Ba L. Peng H.. (2023). The status of e-learning, personality traits, and coping styles among medical students during the COVID-19 pandemic: a cross-sectional study. Front. Psych. 14:1239583. doi: 10.3389/fpsyt.2023.1239583, PMID: 37817833 PMC10561257

[ref31] Zhang Q. Duan H. Liu X. Liu H. Li D. (2020). The current anxiety situation among freshmen in a particular university and its relationship with personality traits. Chin. J. Mod. Med. 3, 69–73,

[ref32] Zhang Y. Tao S. Qu Y. Mou X. Gan H. Zhou P.. (2023). The correlation between lifestyle health behaviors, coping style, and mental health during the COVID-19 pandemic among college students: two rounds of a web-based study. Front. Public Health 10:1031560. doi: 10.3389/fpubh.2022.1031560, PMID: 36711327 PMC9878348

[ref33] Zhao Y. (2021). Investigation on anxiety and coping style of college students during COVID-19 epidemic. Psychiatr. Danub. 33, 651–655. doi: 10.24869/psyd.2021.651 34928925

[ref34] Zung W. W. (1971). A rating instrument for anxiety disorders. Psychosomatics 12, 371–379. doi: 10.1016/S0033-3182(71)71479-0 5172928

