# Supplementary material for: Coping methods of college students with different personality traits when facing COVID-19 from the anxiety psychology perspective
Source: Front Psychol. 2024 May 30;15:1357225. doi: 10.3389/fpsyg.2024.1357225 (PMC11170701; doi:10.3389/fpsyg.2024.1357225)
Supplement: Supplementary file 1 [file Data_Sheet_1.pdf]

Appendix： Survey questionnaire

Basic information

1. Your gender: female, male

2. Your grade: freshman, sophomore, sophomore, junior, and senior

Anxiety Self Rating Scale [Please truthfully fill in the following information based on your recent week's situation.]

Please choose the most suitable item based on your situation: 1->4 indicates very little -->most of the time.

1. I am more nervous and anxious than usual

I will feel scared for no reason

3. I tend to feel restless or scared in my heart

4. I feel like I might be going crazy

5. My hands and feet tremble and tremble

6. I think everything is fine and no misfortune will happen

7. I feel calm and it is easy to sit quietly

8. I feel it is easy to exhale and inhale

I am troubled by headaches, neck pain, and back pain

10. I feel easily weakened and fatigued

11. I feel my heart beating very fast

12. I am troubled by waves of dizziness

13. I have fainting episodes or feel like I am going to faint

14. I feel numb and tingling in my hands and feet

15. I am troubled by stomach pain and indigestion

16. I often have to urinate

My hands and feet are often dry and warm

18. I fall asleep quickly and sleep well all night

19. My face is red and hot

20. I have nightmares

Trait Coping Style Questionnaire

Please choose the most suitable item based on your actual situation: 1->5 means not -->definitely yes

1. Being able to forget unpleasant things quickly
2. Trapped in memories and fantasies about events and unable to get rid of them
3. As if nothing had ever happened
4. It is easy to vent anger on others and often lose your temper
5. Usually think positively and want to be more open-minded
6. Unpleasant things can easily cause emotional fluctuations
7. Keep emotions deep in your heart and not express them, but you cannot forget them
8. Usually, when compared to similar people, one feels that it does not matter much
9. Transforming negative factors into positive ones, such as participating in activities
10. It is easy to feel like crying quietly when encountering troubles
11. Others can easily make you happy again
12. If there is a conflict with someone, it is better to ignore them for a long time
13. Often indecisive and unable to come up with solutions to major difficulties
14. Able to quickly adapt to difficulties and pain
15. Believe that difficulties and setbacks can train people
16. Recalling unpleasant experiences over a long period
17. When encountering difficulties, one often blames oneself for being incompetent and resents oneself
18. Believing that there is nothing major in the world
19. Likes to be alone when facing troubles
20. Usually resolve awkward situations in a humorous way

Eysenck Personality Inventory Simplified Chinese Version

Please choose the most suitable item based on your situation and answer "yes" or "no."

1. Are your emotions fluctuating?

2. Do you feel uncomfortable when you see children (or animals) being tortured?

3. Are you a talkative person?

4. If you say you want to do something, will you always keep your promise regardless of whether it may go smoothly?

5. Do you feel "very miserable" for no reason?

6. Does debt make you feel worried?

7. Are you a vibrant person?

8. Have you ever coveted anything beyond what you deserve?

9. Are you an easily angered person?

10. Do you take drugs that can produce strange or dangerous effects?

11. Are you willing to meet strangers?

12. Have you ever been in a situation where you know you did something wrong but blamed others?

13. Are your emotions easily hurt?

14. Are you willing to act your own way instead of following the rules?

15. Can you keep yourself entertained and have fun at lively gatherings?

16. Are all your habits good?

17. Do you often feel "extremely bored"?

Is good behavior and cleanliness important to you?

19. Are you often proactive when making new friends?

20. Have you ever used to curse people casually?

21. Do you consider yourself a timid and anxious person?

22. Do you think marriage is outdated and should be abolished?

23. Can you easily inject vitality into a dull gathering?

24. Have you ever destroyed or lost someone else's belongings?

25. Are you a worried person?

26. Do you enjoy working with others?

27. Do you tend to stay inconspicuous in social situations?

28. Do you feel worried if you make mistakes in your work?

29. Have you ever spoken ill of or used foul language from others?

30. Do you think you are a person with nervous tension or "strings too tight"?

31. Do you think people spend too much time on savings and insurance for the sake of future security?

32. Do you enjoy spending time with people?

33. When you were still a child, did you ever behave recklessly or disobediently towards your parents?

34. After experiencing an embarrassing event, will you be troubled by it for a long time?

35. Have you made efforts to avoid being rude to others?

36. Do you enjoy having many lively and exciting things around you?

37. Have you ever cheated while playing games?

38. Do you feel pain due to your "neuroticism"?

39. Do you want others to be afraid of you?

40. Have you ever used someone else?

41. Do you enjoy telling jokes and discussing interesting things?

42. Do you often feel lonely?

43. Do you think following social norms is better than acting personally?

44. Are you always full of vitality in the eyes of others?

45. Can you consistently achieve consistency in words and actions?

46. Do you often feel troubled by guilt?

47. Do you sometimes put off what you should do today until tomorrow?

48. Can you make a party go smoothly?
